# Combination of the Auxins NAA, IBA, and IAA with GA_3_ Improves the Commercial Seed-Tuber Production of Potato (*Solanum tuberosum* L.) under *In Vitro* Conditions

**DOI:** 10.1155/2014/439259

**Published:** 2014-06-17

**Authors:** Ahmet Metin Kumlay

**Affiliations:** Department of Field Crops, Faculty of Agriculture, Igdir University, 76000 Igdir, Turkey

## Abstract

The study compared the effects of 1.0 × MS medium containing various concentrations of *α*-naphthaleneacetic acid (NAA), indole-3-acetic acid (IAA), and indole-3-butyric acid (IBA), alone or in combination with gibberellic acid (GA_3_) in micropropagation of three potato (*Solanum tuberosum* L.) cultivars Pasinler, Granola, and Caspar using binodal stem cuttings. The results testified improved regeneration on 1.0 × MS medium containing variants of NAA, IAA, and IBA plus GA_3_ on all cultivars. The minimum days to shoot induction on three cultivars ranged 4.25–5 d on 1.0 × MS medium containing 0.25 mg L^−1^  GA_3_ + 1 mg L^−1^ NAA. The longest shoots (11.8 cm), maximum number of nodes (13.50), and maximum number of leaves (11.00) were recorded on cv. Caspar on 1.0 × MS medium containing 1 mg L^−1^  NAA + 0.25 mg L^−1^ GA_3_. The minimum time to root induction (12.25 d) was noted on cv. Pasinler on the same medium. All of the regenerated shoots could be easily rooted. The results showed that the combined effect of various concentrations of NAA, IAA, and IBA plus GA_3_ was more pronounced compared to the auxins used alone. The results of this research are of significant importance for potato breeders.

## 1. Introduction

The potato (*Solanum tuberosum* L.) is an economically important plant used as food in many countries of the world and also very important plant for the Turkish economy grown over a large area. The potato is the first major food crop in which biotechnology has been successfully implemented for seed production. Previous studies show that micropropagation of potatoes depends on the biological value of cultivars, explant type (leaf, node, shoot tip, etc.), type of culture medium, season, temperature, photoperiod, and a balanced combination of plant growth regulators (PGRs) in the culture media [1–3]. Axillary buds, nodal tissues, stem explants, roots, leaves, tubers, shoots or stems, and cell suspension cultures have been utilized to micropropagate potatoes [1, 4]. The successful* in vitro* multiplication of potatoes depends on the presence of a suitable combination of auxins with gibberellic acid (GA_3_) in the propagation medium [4–7]. Roest and Bokelmann [8] suggested that a lower concentration of auxin with GA_3_ (0.25 mg L^−1^) had a positive impact on the shoot and root development of potato plantlets grown* in vitro*. Ghaffoor et al. [9] have also suggested that the longest shoots were noted on *α*-naphthaleneacetic acid (NAA), the maximum number of nodes on indole-3-butyric acid (IBA), and the maximum number of leaves on indole-3-acetic acid- (IAA-) containing media. Zhang et al. [10] suggest that increased shoot length was promoted among potato explants with increasing concentrations of IAA; however, the stimulating effect of IAA was enhanced by the addition of GA_3_. Badoni and Chauhan [4] showed that the integration of 0.25 mg L^−1^ GA_3_ + 0.01 mg L^−1^ NAA had a positive effect on morphological plantlet characters of cv. Kufri Himalini. Danci et al. [11] obtained the best results from 1.0 × MS media containing 1 mg L^−1^ IAA, 1 mg L^−1^ IBA, and 0.3 mg L^−1^ GA_3_ from Amelia, Cristian, Nicoleta, and Roclas potato cultivars.

Since each individual hormone has its own unique effect on regeneration, it is vital to determine the combined effects of these on the regeneration of shoots and roots. Therefore, the present study aimed to determine the best combination of NAA, IBA, and IAA, used singly or in combination with GA_3_, for successful* in vitro* commercial seed-tuber production of potato cvs. Pasinler, Granola, and Caspar using meristem-derived stem node cultures.

## 2. Materials and Methods

### 2.1. The Concentrations of PGRs and Media Preparation

1.0 × MS medium (1962) containing 0.25 mg L^−1^ GA_3_, 1 mg L^−1^ NAA, 1 mg L^−1^ IAA, and 1 mg L^−1^ IBA with and without 0.25 mg L^−1^ GA_3_ complemented with 3% (w/v) sucrose and 0.8% (w/v) agar was used. The pH was calibrated to 5.6–5.8 by 1 N HCl or 1 N NaOH after adding all medium components except the agar. GA_3_, IAA, and IBA are thermolabile; therefore, they were filter-sterilized by passing through 0.2 *μ*m Millipore filters (Schleicher & Schuell, FP 30/0.2 CA-S; 0.2 *μ*m; 7 bar max) inside a laminar flow cabin before adding to each of culture medium after autoclaving at 45°C. The cultures were sterilized by autoclaving at 120°C for 20 min and 104 kPa pressure.

### 2.2. Plant Material and Micropropagation of Explants

Binodal explants from three potato cultivars, namely, Pasinler (locally improved and registered mid-early maturing cultivar), Granola (mid-late-maturing cultivar), and Caspar (late-maturing cultivar), were used in the present study. Binodal cuttings were aseptically cultured on 1.0 × MS medium containing 10 binodal explants per replication and 40 binodal explants per treatment. Each replication consisted of one glass jar and all experiments were replicated four times. Cultures were incubated at a temperature of 24 ± 2°C under 2,000 lux light intensity with 16 h of day light photoperiod for 6 weeks.

### 2.3. Statistical Analysis

The data were recorded for mean regeneration percentage (%), days to shoot and root induction, shoot and root length (cm), and the number of nodes, leaves, and roots per shoot. A completely randomized design (CRD) was used to evaluate the three cultivars, eight plant growth regulator combinations, and four replications. Data were subjected to analysis of variance and the means were separated by Duncan's multiple range test. Results on all parameters are the means and standard errors (±SE) from four replications.

## 3. Results and Discussion

The effect of variants of auxins plus GA_3_ on the binodal explants regeneration rate, shoot and root length, days to shoot and root induction, number of nodes, leaves and roots were significantly different (*P* < 0.01). The interaction between treatments and cultivar potentiality for the length of shoots and roots, the number of nodes and leaves, and root induction days also showed significant variations (*P* < 0.01). The results of each treatment and cultivar interaction are presented below under their individual subheadings.

### 3.1. Regeneration Rate

**Figure 1:**
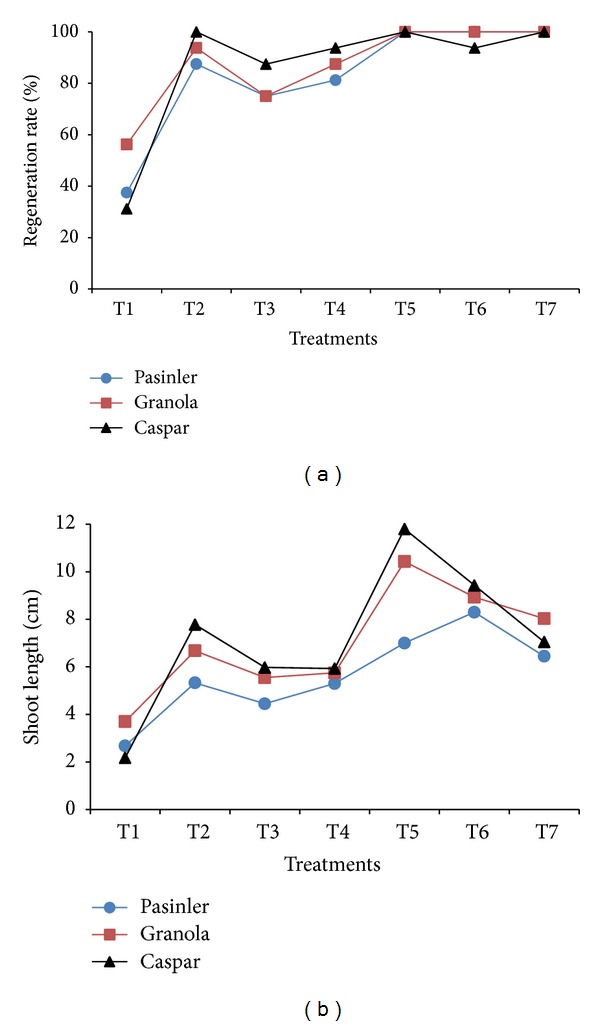
Effects of 1.0 × MS medium containing 1 mg L^−1^ of NAA, IAA, and IBA, with and without 0.25 mg L^−1^ GA_3_, on the regeneration rate and shoot length of cvs. Pasinler, Granola, and Caspar. Means of different values from four replications (*n* = 4) are statistically different using Duncan's multiple range test at the 0.01 level of significance. (T1: 0.25 mg L^−1^ GA_3_, T2: 1 mg L^−1^ NAA, T3: 1 mg L^−1^ IAA, T4: 1 mg L^−1^ IBA, T5: 0.25 mg L^−1^ GA_3_ + 1 mg L^−1^ NAA, T6: 0.25 mg L^−1^ GA_3_ + 1 mg L^−1^ IAA, T7: 0.25 mg L^−1^ GA_3_ + 1 mg L^−1^ IBA.)

PGRs combinations in 1.0 × MS medium affected the regeneration of binodal explants variably (*P* < 0.01). Cent percent regeneration (100%) of the three cultivars was observed on agar-solidified 1.0 × MS medium containing each of 1 mg L^−1^ NAA, IAA, and IBA with 0.25 mg L^−1^ GA_3_. However, 1.0 × MS medium containing NAA, IAA, IBA, and 0.25 mg L^−1^ GA_3_ singly was inhibitory and produced a reduced regeneration rate (Figure 1). These results are in agreement with a study by Webb et al. [12] in which they suggested that the sequential application of hormones (IAA and NAA), in the presence of GA_3_, enhanced shoot regeneration from the leaf discs of explants of six potato cultivars. Miller et al. [13] using the slow-growing Desiree, Record, Foxton, and Golden Wonder cultivars found that a combination of 1 mg L^−1^ GA_3_ and 0.1 mg L^−1^ NAA effectively increased the number of nodes which could be cultured thereafter. The results of this study are also similar to those of Badoni and Chauhan [4], which suggested that 0.01 mg L^−1^ NAA with 0.25 mg L^−1^ GA_3_ was the best substrate for shoot regeneration in the potato cultivar “Kufri Himalini.”

### 3.2. Shoot Length

Variants of NAA, IAA, and IBA plus GA_3_ affected shoot length variably, showing significant differences among them (*P* < 0.01). The longest shoots were noted on cv. Caspar (11.8 cm) using 0.25 mg L^−1^ GA_3_ + 1 mg L^−1^ NAA followed by Granola (10.43 cm) on the same medium, and Caspar (9.43 cm) using 1.0 × MS medium containing 0.25 mg L^−1^ GA_3_ + 1 mg L^−1^ IAA. The minimum shoot length was recorded on cv. Caspar (2.18 cm) using 1.0 × MS medium containing 0.25 mg L^−1^ GA_3_ (Figure 1). The results in line with findings of Webb et al. [12] testified that IAA and NAA, in combination with GA_3_, enhanced shoot elongation. Zhang et al. [10] suggested that the GA_3_ and IAA positively affected the shoot length in cv. Zihubai. Farhatullah and Sayeed [14] obtained 9.1 cm long shoots on 0.248 mg L^−1^ GA_3_. Badoni and Chauhan [4, 6] found that the combination of 0.25 mg L^−1^ GA_3_ + 0.01 mg L^−1^ NAA concentrations increased shoot length. In contrast, O. M. Danci and M. Danci [2] and Hoque [7] observed the best shoot regeneration on IAA, and Shibli et al. [15] and Al-Taleb et al. [16] obtained their best results on MS medium containing IBA.

### 3.3. Days to Shoot Induction

**Figure 2:**
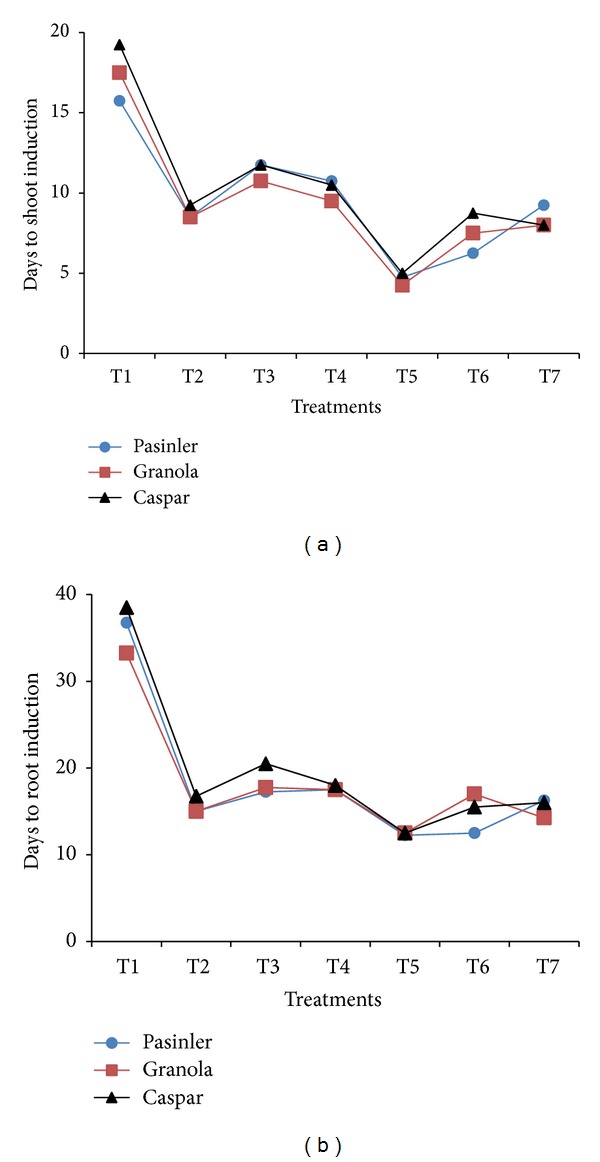
Effects of 1.0 × MS medium containing 1 mg L^−1^ of NAA, IAA, and IBA, with and without 0.25 mg L^−1^ GA_3_, on the number of days to shoot and root induction of cvs. Pasinler, Granola, and Caspar. Means of different values from four replications (*n* = 4) are statistically different using Duncan's multiple range test at the 0.01 level of significance. (T1: 0.25 mg L^−1^ GA_3_, T2: 1 mg L^−1^ NAA, T3: 1 mg L^−1^ IAA, T4: 1 mg L^−1^ IBA, T5: 0.25 mg L^−1^ GA_3_ + 1 mg L^−1^ NAA, T6: 0.25 mg L^−1^ GA_3_ + 1 mg L^−1^ IAA, and T7: 0.25 mg L^−1^ GA_3_ + 1 mg L^−1^ IBA.)

The effect of all PGR applications on shoot induction days was significantly different (*P* < 0.01). The minimum days to shoot induction were noted on cv. Granola (4.25 d), followed by cv. Pasinler (4.75 d) and cv. Caspar (5.00 d) on 1.0 × MS medium containing 0.25 mg L^−1^ GA_3_ + 1 mg L^−1^ NAA. However, late-maturing cv. Caspar had the most delayed shoot induction period of 19.25 d, followed by 17.50 on cv. Granola and 15.75 d on cv. Pasinler on 1.0 × MS medium containing 0.25 mg L^−1^ GA_3_ (Figure 2). The results show the experimental treatments were effective to reduce time to shoot induction compared to Yasmin et al. [17], who noted the minimum days to shoot induction on cvs. Desiree (4.3 d) and Patrones (5.1 d) using 0.5 mg L^−1^ GA_3_ + 1 mg L^−1^ pantothenic acid. Although the minimum number of days to shoot induction was determined on 1.0 × MS medium containing 0.25 mg L^−1^ GA_3_ + 1 mg L^−1^ NAA in the present study, Hoque [7] observed that IAA-containing media accelerate time to regenerate with the least time for shoot regeneration.

### 3.4. Days to Root Induction

Statistically significant effects were recorded for all PGR applications on the number of days to root induction (*P* < 0.01). The minimum number of days required for root induction was noted on 1.0 × MS medium containing 0.25 mg L^−1^ GA_3_ + 1 mg L^−1^ NAA (12.25 d for cv. Pasinler) and 12.50 d each for cv. Granola and cv. Caspar (Figure 2). The treatment shown was significantly effective in decreasing the days to root induction compared to the previous reports, where Yasmin et al. [17] observed the least number of 16.7 d to root induction in cv. Desiree and 25.9 d in cv. Patrones potato meristems by using 0.5 mg L^−1^ GA_3_ + 1 mg L^−1^ pantothenic acid and control, respectively.

### 3.5. Number of Nodes

**Figure 3:**
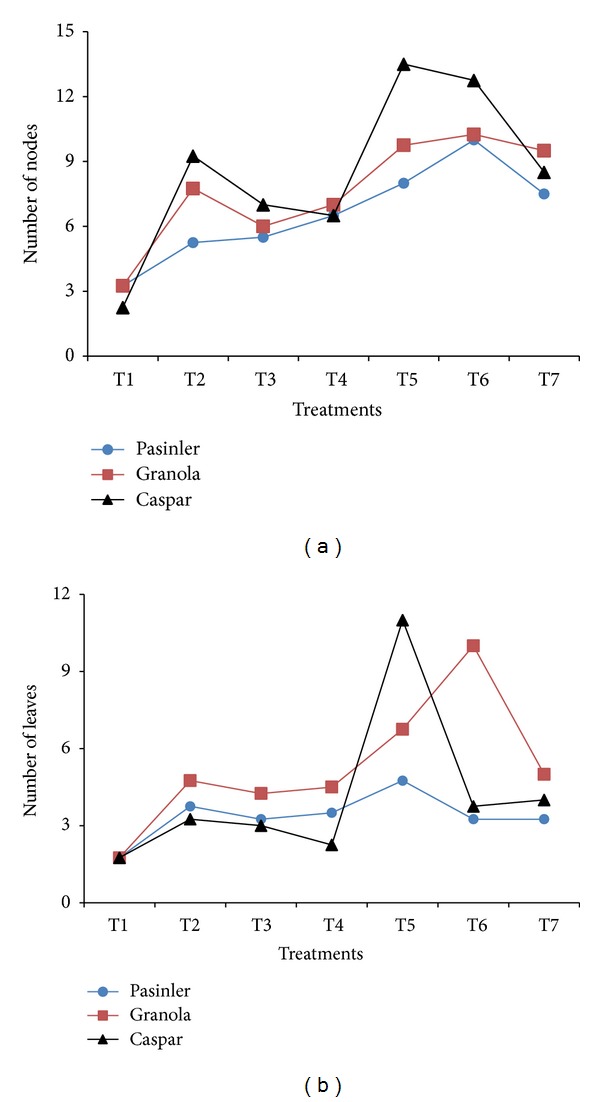
Effects of 1.0 × MS medium containing 1 mg L^−1^ of NAA, IAA, and IBA, with and without 0.25 mg L^−1^ GA_3_, on the number of nodes and leaves of cvs. Pasinler, Granola, and Caspar. Means of different values from four replications (*n* = 4) are statistically different using Duncan's multiple range test at the 0.01 level of significance. (T1: 0.25 mg L^−1^ GA_3_, T2: 1 mg L^−1^ NAA, T3: 1 mg L^−1^ IAA, T4: 1 mg L^−1^ IBA, T5: 0.25 mg L^−1^ GA_3_ + 1 mg L^−1^ NAA, T6: 0.25 mg L^−1^ GA_3_ + 1 mg L^−1^ IAA, and T7: 0.25 mg L^−1^ GA_3_ + 1 mg L^−1^ IBA.)

Plant growth regulators affected the number of nodes per binodal explant significantly (*P* < 0.01). The maximum number of nodes was obtained on cv. Caspar on 1.0 × MS medium containing 0.25 mg L^−1^ GA_3_ + 1 mg L^−1^ NAA (13.50) and 0.25 mg L^−1^ GA_3_ + 1 mg L^−1^ IAA (12.75). The minimum nodes on each cultivar were obtained on 1.0 × MS medium containing only 0.25 mg L^−1^ GA_3_ (Figure 3). The results are improvement over previous reports by Badoni and Chauhan [4], Badoni and Chauhan [6], Miller et al. [13], Hassan et al. [18], and Zaman et al. [19]. Miller et al. [13] noted that the combination of 1 mg L^−1^ GA_3_ + 0.1 mg L^−1^ NAA was effective in increasing the number of nodes (7.6). Zaman et al. [19] reported that a higher concentration of auxins resulted in a higher number of nodes (7.3). Badoni and Chauhan [4, 6] found that the 0.25 mg L^−1^ GA_3_ + 0.01 mg L^−1^ NAA concentrations increased the number of nodes (both 9.4 nodes). Ghaffoor et al. [9] obtained a higher number of nodes (9.7) on MS medium containing IBA. The results of Shibli et al. [15] showed that the total number of nodes ranged from 10.2 (at 2.0 mg L^−1^ IBA + 1 mg L^−1^ GA_3_) to 3.5 nodes/test tube (at 2.0 mg L^−1^ IAA + 1 mg L^−1^ GA_3_). In another study, Armin et al. [20] also speculated that the application of NAA completely inhibited the growth of single nodes of* in vitro* grown potato plantlets.

### 3.6. Number of Leaves

The number of leaves per binodal explant showed significant variation (*P* < 0.01) after treatment with 1.0 × MS medium containing 1 mg L^−1^ NAA, 1 mg L^−1^ IAA, and 1 mg L^−1^ IBA, with and without 0.25 mg L^−1^ GA_3_. The maximum number of leaves per binodal explant on cv. Caspar (11.00) and on cv. Granola (10.00) was obtained on 0.25 mg L^−1^ GA_3_ + 1 mg L^−1^ NAA and on 0.25 mg L^−1^ GA_3_ + 1 mg L^−1^ IAA, respectively, which was followed closely by 6.75 leaves per explant on cv. Granola using 0.25 mg L^−1^ GA_3_ + 1 mg L^−1^ NAA. The minimum leaves per explant (1.75) on each of the three cultivars were noted on 1.0 × MS medium containing 0.25 mg L^−1^ GA_3_ (Figure 3). The results have edge over previous results with more number of leaves per explant. Farhatullah and Sayeed [14] reported the maximum number of leaves (7.3) on 0.248 mg L^−1^ GA_3_. Similarly, Zaman et al. [19] regenerated maximum number of leaves (8.9) using 0.5 mg L^−1^ NAA. Ghaffoor et al. [9] regenerated the maximum number of 6.143 leaves per explant on 0.25 mg L^−1^ IBA containing 1.0 × MS medium.

### 3.7. Number of Roots

**Figure 4:**
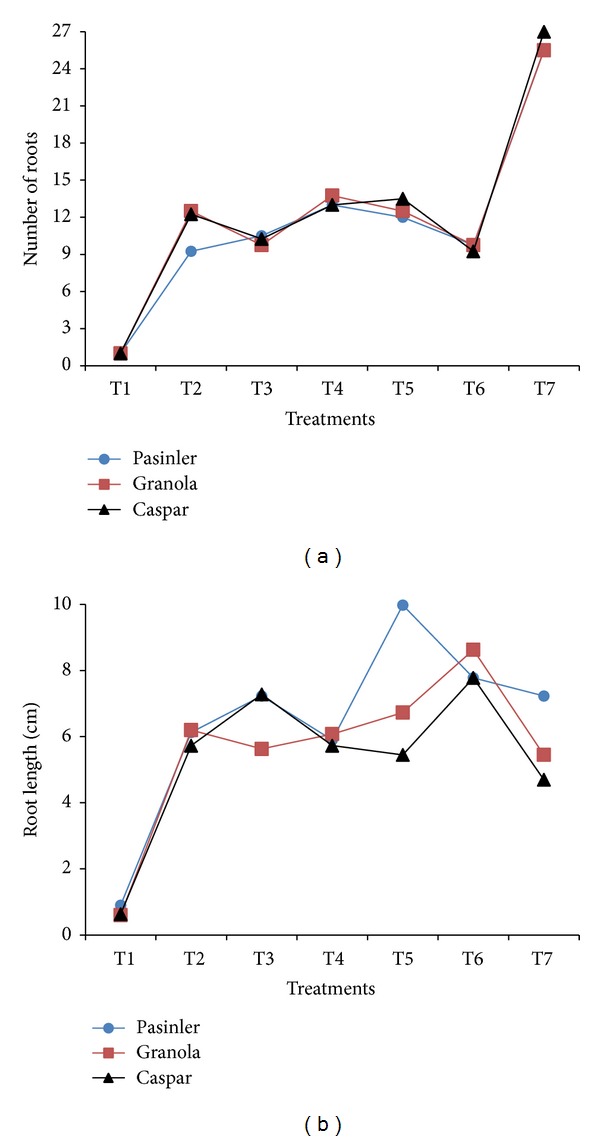
Effects of 1.0 × MS medium containing 1 mg L^−1^ of NAA, IAA, and IBA, with and without 0.25 mg L^−1^ GA_3_, on the number of roots and root length of cvs. Pasinler, Granola, and Caspar. Means of different values from four replications (*n* = 4) are statistically different using Duncan's multiple range test at the 0.01 level of significance. (T1: 0.25 mg L^−1^ GA_3_, T2: 1 mg L^−1^ NAA, T3: 1 mg L^−1^ IAA, T4: 1 mg L^−1^ IBA, T5: 0.25 mg L^−1^ GA_3_ + 1 mg L^−1^ NAA, T6: 0.25 mg L^−1^ GA_3_ + 1 mg L^−1^ IAA, and T7: 0.25 mg L^−1^ GA_3_ + 1 mg L^−1^ IBA.)

There was a significant (*P* < 0.01) effect of all PGR applications on the number of roots. The maximum number of roots was observed on cv. Caspar (27.00), followed by cv. Pasinler (25.50) and cv. Granola (25.50) on medium containing 0.25 mg L^−1^ GA_3_ + 1 mg L^−1^ IBA. The minimum roots (1.0) grew on medium containing 0.25 mg L^−1^ GA_3_ (Figure 4). The results presented here are improvement over the previous results. Sanavy and Moeini [21] suggested that the application of 1.5 mg L^−1^ NAA decreased the number of roots from 5 to 3. In another study, Al-Taleb et al. [16] recorded the highest the number of 10.40 roots per explant from cv. Spunta on media containing IBA. Zaman et al. [19] reported 23.7 roots on 1 mg L^−1^ IBA containing medium. Shibli et al. [15] obtained the best results (16.2 roots) from IBA-containing media. Hoque et al. [7] obtained their maximum number of roots (17.4) using 0.25 mg L^−1^ IAA. Dhital et al. [3] observed that 1.0 mg L^−1^ NAA gave rise to a greater number of roots (9.5) than 1.0 mg L^−1^ IAA (4.0). Uddin [5] determined the highest number of roots (4.4) on 0.5 mg L^−1^ IBA-containing medium.

### 3.8. Root Length

The PGR combinations affected the root length significantly (*P* < 0.01). The longest roots were noted on cv. Pasinler (9.98 cm) on 0.25 mg L^−1^ GA_3_ + 1 mg L^−1^ NAA, followed by cv. Granola (8.63 cm) and cv. Caspar (7.78 cm) on 1.0 × MS medium containing 0.25 mg L^−1^ GA_3_ + 1 mg L^−1^ IAA. The minimum root length on each cultivar was determined to be 0.90, 0.60, and 0.63 cm for cvs. Pasinler, Granola, and Caspar, respectively, on 1.0 × MS medium containing 0.25 mg L^−1^ GA_3_ (Figure 4). Zaman et al. [19], Sanavy and Moeini [21], and Uddin [5] emphasize that an increase in root length and number is very important for acclimatization to* ex vitro* conditions, as well as water and nutrient uptake in potato plantlets. The results are improvement over the results of Farhatullah and Sayeed [14], who reported their longest roots (3.7 cm) on 0.248 mg L^−1^ GA_3_ containing MS medium. The present results are in agreement with those of Badoni and Chauhan [4, 6], who found that the combination of 0.25 mg L^−1^ GA_3_ + 0.01 mg L^−1^ NAA increased root length (11.9 cm). However, several researchers have reported that the longest roots were grown on IAA [2, 7, 22] and on IBA-containing medium [16]. Sanavy and Moeini [21] illustrated that the application of NAA decreased the length of potato roots from 6 cm on control to 4 cm on 1.5 mg L^−1^ NAA containing MS medium. Zaman et al. [19] reported the longest roots (4.2 cm) from 1 mg L^−1^ IAA. Haque et al. [22] showed potato explants produced better results for root length on 1.0 mg L^−1^ IAA + 0.25 mg L^−1^ GA_3_ (7.38 cm).

## 4. Conclusion

Multiplication of potatoes* in vitro* has proven to be a very efficient technique to accelerate the production of high quality, healthy plantlets, in terms of genetic and physiological uniformities with high photosynthetic potential. It has been shown that when conditions are available for regeneration, shoots, roots, and stem explants with node(s) can regenerate easily, even in the absence of any PGRs. However, the presence of PGRs would lengthen the regeneration time and decrease the number of shoots, nodes, and roots produced, which inhibits the production of healthy seed potatoes [23, 24]. Adding exogenous GA_3_ with different auxins is a good way to reduce micropropagation time and increase the number of plantlets for* in vitro* micropropagation of potatoes [25]. These PGRs are of great importance in regulating shoot and root development in potatoes* in vitro* [14]. This study reports shoot regeneration from binodal explants of potato for the first time. The results presented here show improvement over previous results in general terms using different explants obtained from various cultivars [4, 6, 7, 17, 19].

Cultivars showed wide variation in their response to PGRs and a genotype-dependent response to a combination of GA_3_ and auxins for the multiplication of cvs. Pasinler, Granola, and Caspar. The results of this study show a general improvement compared to the results of other studies and suggest that the effects of NAA, IAA, and IBA with GA_3_ were more pronounced than regeneration on media containing NAA, IAA, or IBA singly for* in vitro* micropropagation of three potato cultivars. Therefore, appropriate concentration of GA_3_ with the auxins is essential for direct and efficient regeneration of binodal explants without callus formation and normal axillary shoot growth* in vitro*. The results of this study show edge over previous studies in all of the studied parameters [26–28]. It may be concluded that among the seven different PGR treatments, 1.0 × MS medium containing 1 mg L^−1^ NAA + 0.25 mg L^−1^ GA_3_ improved the micropropagation capacity of the three cultivars studied and resulted in the maximum improvement in the parameters. It should also be noted that the control treatment lacking auxins had an inhibitory effect on all of the studied plantlet characteristics. This protocol meets the objectives of the study and provides solid basis for the commercial mass production of the studied cultivars through* in vitro* micropropagation techniques.
